# An NMR Study on Hydration and Molecular Interaction of Phytantriol-Based Liquid Crystals

**DOI:** 10.3390/pharmaceutics14112312

**Published:** 2022-10-27

**Authors:** Yu Lu, Di Zhu, Quynh Le, Yuji Wang, Wei Wang

**Affiliations:** 1 Department of Medicinal Chemistry, College of Pharmaceutical Sciences, Capital Medical University, Beijing 100069, China; 2 Beijing Area Major Laboratory of Peptide and Small Molecular Drugs, Engineering Research Center of Endogenous Prophylactic of Ministry of Education of China, Beijing 100069, China; 3 Department of Chemistry, University of Bergen, P.O. Box 7803, 5020 Bergen, Norway; 4 Center for Pharmacy, University of Bergen, P.O. Box 7803, 5020 Bergen, Norway

**Keywords:** phytantriol, LLCs, NMR, NOESY, hydration, molecular interaction

## Abstract

Phytantriol-based lyotropic liquid crystals (LLCs) have emerged as a new nanodrug delivery system. However, the understanding of phytantriol-based LLCs is lacking. In this study, we use NMR technology to characterize LLC formation over two months. Three samples in different phases were prepared with different hydration states. NMR data, including 1D-^1^H, ^13^C-{^1^H}, 2D-HSQC, HMBC, COSY, NOESY, etc., were collected. A comprehensive analysis of these NMR data was performed on the three phases of phytantriol-based LLCs. The following results were achieved from the study. First, the ^1^H and ^13^C-{^1^H} spectra of phytantriol were assigned. Second, the change of NMR spectra during the formation of the phases was observed, and the change of hydration was calculated for the time-dependent phase formation. Third, the correlation peaks of 2D-NOESY were used to describe the spatial relationship of lipids–water interaction and lipid–lipid interaction.

## 1. Introduction

**Figure 1:**
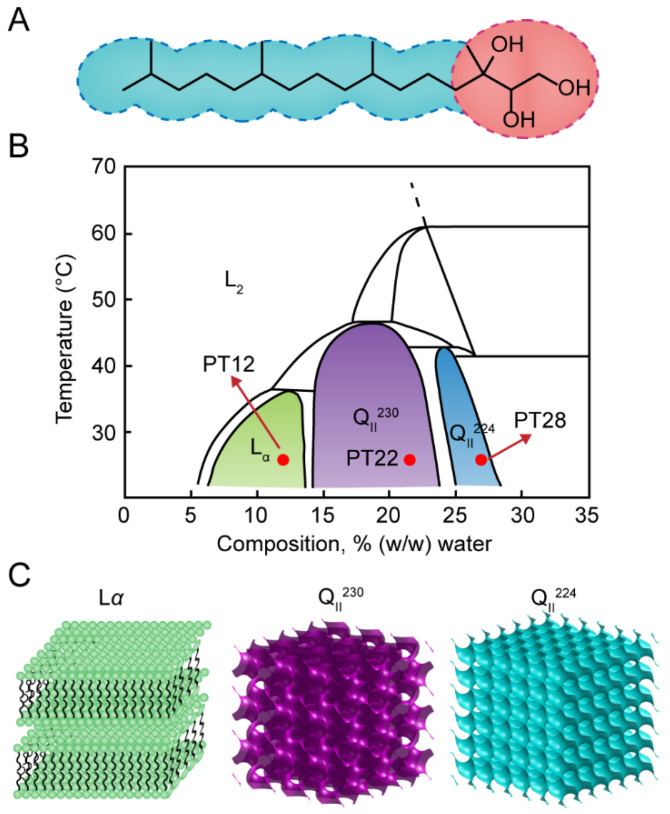
The introduction of phytantriol-based LLCs. (**A**) The structure of phytantriol molecule. (**B**) The phase diagram of the phytantriol/water system. (**C**) 3D structure of Lα, Q_II_^230^ and Q_II_^224^ phase.

The lyotropic liquid crystals (LLCs) are a system of amphiphilic molecules formed spontaneously in a solvent [1]. According to the difference of the arrangement of surfactant aggregates, the surfactants form different phases, including layered phase (Lα), hexagonal phase (H_II_), inverse cubic phase (Q_II_), and etcetera [2]. The formation of these phases is closely related to the water content and temperature of the system. Phytantriol (3,7,11,15-tetramethylhexadecane-1,2,3-triol, PHYT) (Figure 1A) is one of the frequently used amphiphilic molecules which forms the reversed cubic phase (Figure 1B,C). In a different hydration state, PHYT forms Q_II_, Lα, and L_II_ phases (Figure 1B,C). For example, in low water content (6–14% wt), PHYT forms the L_II_ phase at room temperature. With the increase water content, the L_II_ phase transfers to the Q^230^ cubic phase with Ia3d crystallographic space group symmetry. PHYT arranges itself on a gyroid surface to minimize contact with water. With a further increase of water content to 25–30%wt, PHYT forms the Q^224^ cubic phase with Pn3m crystallographic space group symmetry and PHYT self-assembles on a diamond surface.

Water channels in the Q_II_, Lα, and L_II_ phases could be used to encapsulate hydrophilic drugs, and the lipid bilayer can be used to encapsulate hydrophobic drugs [3]. Therefore, the applications of phytantriol-based LLCs are realized in drug delivery systems, such as transdermal preparations [4], sustained-release preparations [5,6], Ocular drug delivery [7], and oral preparations [8].

Nuclear magnetic resonance (NMR) spectroscopy is of great significance for the characterization of LLCs [9]. By comparing the ^1^H and ^13^C-{^1^H} spectra of LLCs with ordinary solution NMR, isotropic and anisotropic LLCs could be easily distinguished according to the chemical shift anisotropy (CSA) effect [10]. At the same time, the hydration of LLCs system can be calculated according to the integration of water peak in the ^1^H spectra [11]. In addition, by comparing NMR over time, dynamic behaviors occurring during phase formation—such as lipid reorientation as water molecules penetrate—can be monitored [12,13,14,15].

At present, the NMR studies of reversed cubic LLCs mainly focus on monoolein-based LLCs [16,17]. So far, we have not found the NMR studies on phytantriol-based LLCs. Therefore, the purpose of the current study was to investigate phytantriol-based LLCs with different hydration levels. As indicated in the phase diagram, the hydration level is decisive to its phase behavior; therefore, three representative samples were prepared for the NMR study. The phytantriol-based LLCs were comprehensively analyzed through the characterization of multiple NMR spectra (^1^H, ^13^C-{^1^H}, NOESY, COSY, HSQC, and HMBC). The following questions are therefore answered after reading the study: (1) the ^1^H and ^13^C-{^1^H} spectra of phytantriol could be assigned; (2) the time-dependent NMR spectra were analyzed, and the change of hydration with time could correlate to the phase transition during phase formation; (3) the correlation peaks of two-dimensional NOESY revealed the spatial relationship of a lipid interaction and a lipid–water interaction.

## 2. Materials and Methods

### 2.1. Preparation of NMR Samples

The phytantriol used in this study was purchased from Avanti Polar Lipids (SKU 850556O). The water used was purified milli-Q water. Deuterium chloroform was purchased from Sigma-Aldrich (151823). A certain amount of phytantriol was weighed into a 3 mm diameter inner nuclear tube (Bruker biospin, 4″ NMR-Tubes, 3 mm, Z172597) and then mixed with water. The amount of PHYT and water for each sample are listed in Table S1. After flame sealing, the sample was centrifuged up and down at 4000× *g* for 60 min per inversion, and the centrifugation was repeated several times according to the mixing degree. Then, the sample tube (3 mm) was added into a 5 mm NMR tube (Bruker biospin, 4″ NMR-tubes, 5 mm, Z172599) and filled with 300 μL CDCl_3_. CDCl_3_ in the 5 mm NMR tube was used for the field-frequency locking. The samples were kept at room temperature for approximately 12 h before the first NMR measurement. Then, they were stored in NMR tube racks at 298 K for the remainder of the measurements.

### 2.2. NMR Spectroscopy Acquisition

All NMR data were obtained on a Bruker Ascend 600 MHz spectrometer equipped with an AVANCE NEO console. The probe was a QCI-P CryoProbe. For ^1^H, the Larmor frequency was 600.13 MHz; for ^13^C, it was 150.93 MHz. The settings and parameters used in the experiments are specified in Table S2. All NMR spectral data were analyzed using MestRenova (v14). The ^1^H chemical shift of CDCl_3_ was at 7.260 ppm, which was used as a reference. The ^13^C-{^1^H} chemical shift of CDCl_3_ was at 77.160 ppm, also used as a reference.

### 2.3. NMR Data Analysis

The hydration of samples was calculated by ^1^H NMR spectra, and all spectra were collected and processed under the same experimental parameters. The multiplets module of MestRenova was used to integrate the water peak and phytantriol peaks. Then, the hydration of different samples was calculated using the following equations:

(1) $$I_{w}=M\;\cdot \;\frac{{X^{\prime }}_{w}}{9}$$

(2) $$I_{lip}=N_{lip}\;\cdot M\;\cdot \;\frac{1-Xw^{\prime }}{M_{lip}}$$

In these equations, *I_w_* and *I_lip_* are the integrals of water and phytantriol, respectively, obtained directly from the ^1^H NMR spectrum. *M* is the total weight of the sample, and *X_w_*′ is the weight percentage of water in the sample. *N_lip_* and *M_lip_* are the number of protons and their molecular weight in phytantriol, respectively. *N_lip_* = 42 and *M_lip_* = 330.

By dividing Equations (1) and (2), the relationship between the ratio of the water peak integral to the lipid peak integral (*I_w_*/*I_lip_*) and *X_w_*′ can be obtained. Then, the water peak integral was set to 1, and *X_w_*′ can be calculated.

## 3. Results and Discussion

### 3.1. Chemical Shift Attribution

**Figure 2:**
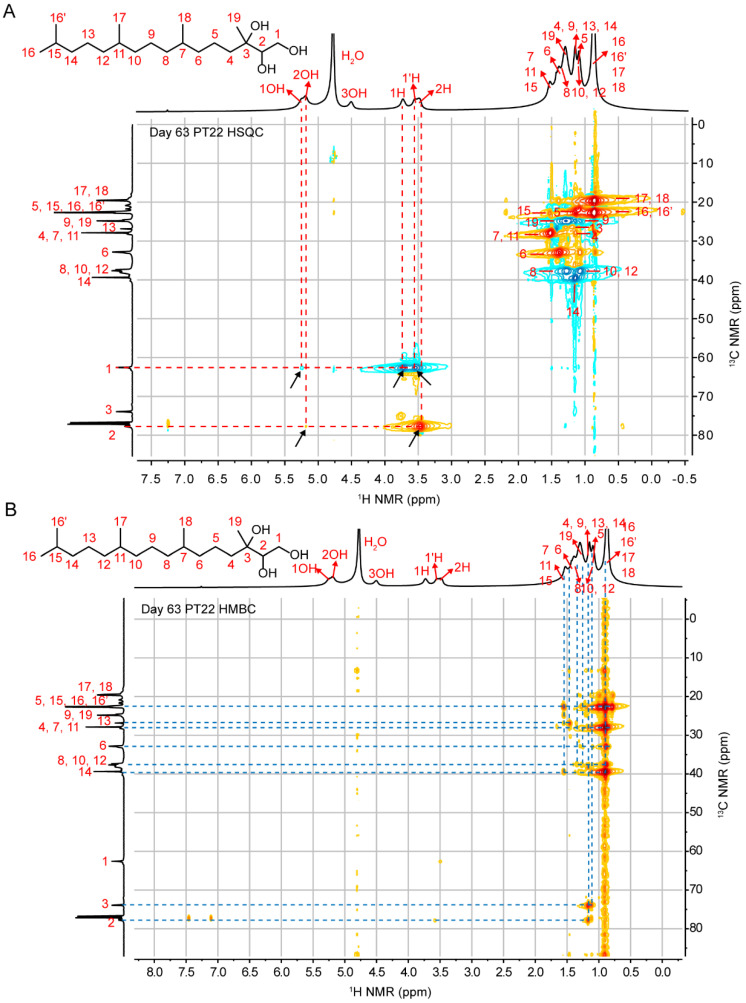
(**A**) ^1^H—^13^C HSQC spectrum and (**B**) remotely coupled ^1^H—^13^C HMBC spectrum of PT22 (22% wt H_2_O) acquired at day 63.

Through a comprehensive analysis of the ^1^H NMR, ^13^C-{^1^H} NMR, ^1^H-^1^H COSY, ^1^H-^13^C HSQC and ^1^H-^13^C HMBC spectra in Figure 2 and Figure S1 for PT22 at day 63, the ^1^H and ^13^C-{^1^H} peaks of all hydrogen and carbon in phytantriol molecules were assigned for the first time in this study. First, through the correlation analysis of adjacent C-H (^1^J_C-H_) in the HSQC spectrum, we found that C3 had no correlation with 1–3 OH, so the position of C3 was inferred to be 73.865 ppm. Secondly, according to the correlation between C1 and two hydrogens (1H and 1H′), the position of C1 was 62.570 ppm. The remaining peak in the low field region of the carbon spectrum was C2. Meanwhile, we also ascribed peak positions of 1OH, 2OH, 3OH, 1H, 1′ H and 2H in the ^1^H spectrum (Figure 2A). Similarly, together with the correlation analysis of C-H (*^n^J*_C-H_, *n* = 2, 3, 4) above the two chemical bonds in HMBC spectrum, we performed the preliminary attribution of alkane H between 1.5–0.5 ppm and alkane C between 40–15 ppm (Figure 2B). The attribution of ^1^H and ^13^C-{^1^H} peaks is summarized in Table 1.

### 3.2. Hydration of Phytantriol/Water LCP

**Figure 3:**
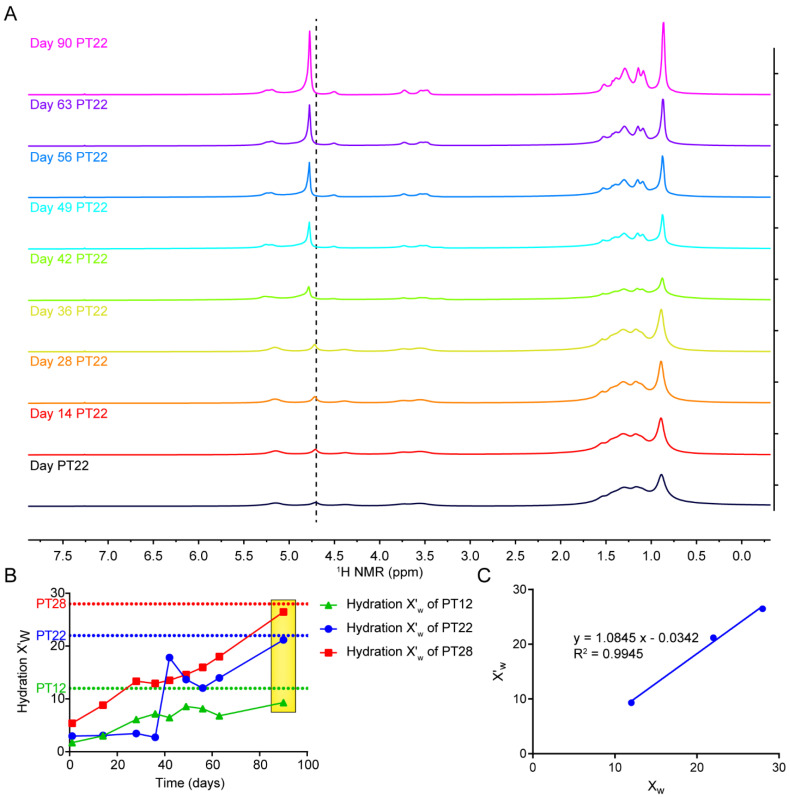
The hydration of phytantriol/water LCP. (**A**) ^1^H NMR spectra of PT22 changed with time. (**B**) hydration level of PT12, PT22 and PT28 changed with time (on the day 1, 14, 28, 36, 42, 49, 56, 63 and 90). (**C**) The relationship between actual hydration and theoretical value on day 90.

**Figure 4:**
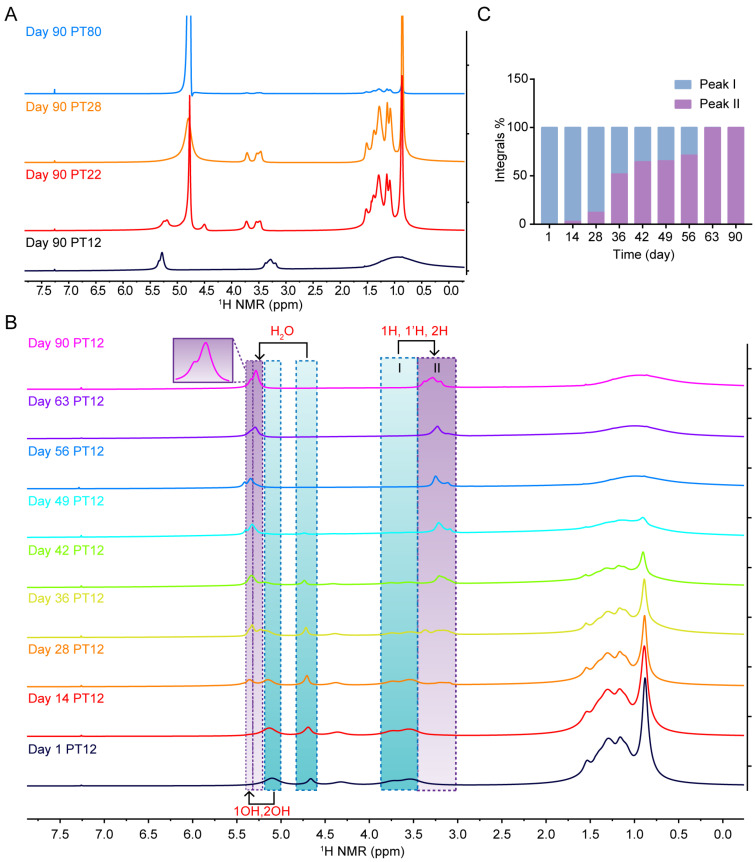
Isotropy and anisotropy of LCP. (**A**) ^1^H spectra of LCP and solution with different hydration degree (12 wt %, 22 wt %, 28 wt %, 80 wt %). (**B**) ^1^H NMR spectra of PT12 changed with time. (**C**) The integral proportions (%) between peak I and peak II.

NMR could provide a unique perspective for the study of dynamic changes of the lipid crystal phase (LCP) [18], and the change of hydration level of LCP can be revealed by ^1^H resonance integration [19,20]. So far, there is no similar study on the determination of the hydration of phytantriol/water LCP by NMR. ^1^H spectra of phytantriol/H_2_O LCP with different hydration were measured continuously for 90 days, and the actual hydration was calculated according to Equations (1) and (2). In Figure 3A, Figure 4A and Figure S3, ^1^H spectra of the three samples with different hydration changed over time. Specifically, the resonance intensity of water increased gradually with time, indicating that the water content in the sample increased gradually (Figure 3B). At the same time, after 90 days of storage at 25 °C, the relationship between the calculated hydration value (X_w_′) and the theoretical value (X_w_) was shown in Figure 3C, and the R^2^ value of linear correlation between them was 0.9945, indicating that the ^1^H spectrum could accurately determine the hydration level of phytantriol/H_2_O LCP. In addition, with the increase of water compatibility, the peak of water gradually shifted to a lower field.

### 3.3. Isotropy and Anisotropy of LCP

According to the previously reported phase diagram of the phytantriol/water system, PT12 is in L*α* phase, PT22 in Q^230^ phase, and PT28 in Q^224^ phase [21]. In Figure 4A, the peak shapes in ^1^H spectra of PT22 and PT28 were similar to that of ordinary aqueous phytantriol PT80, indicating that PT22 and PT28 samples were isotropic LCP. However, the spectral chemical shift of PT12 was completely different from that of PT80, and peak broadening existed, indicating that PT12 is anisotropic LCP. All spectral differences of these phases indicate that the spectra of the isotropic phase can clearly show the peak coupling, while the spectra of the anisotropic phase shows an unsplit single peak [22].

We further observed the changes in the ^1^H spectra of PT12 with time in Figure 4B. On the first day, the spectrum was similar to that of the other samples, because the lipid molecules were randomly distributed in the system and exhibited an isotropic behavior [22]. However, as the experiment proceeded, two types of OH peaks (water peak and 1H and 2H peaks) were observed in ^1^H spectra. In particular, the peaks of 1H and 2H gradually shifted from the position of peak I to the position of peak II. This result suggested that the phytantriol molecules started self-assembling due to slow diffusion over time, redirecting to the anisotropic Lα phase [17,23]. The integrals of peak I and peak II in ^1^H spectra were calculated (Table 2) and the changes in the integral proportion were obtained in Figure 4C. The proportion of peak II can reflect the formation of anisotropic Lα. The anisotropy Lα phase was gradually formed until day 63. The doublet splitting may suggest the coexistence of two phases during 14 to 56 days [24,25,26].

The ^13^C-{^1^H} NMR spectra (Figures S4–S6) had similar trends with ^1^H spectra over time [15]. Specifically, the C peaks of PT12 (Figure S4) were shifted and widened substantially, possibly due to the phase formation of L*α*. The chemical shifts of carbon were affected by changes in the bilayer/water interface. Unlike the changes in the ^1^H spectrum (where only the chemical shifts of H in the head groups changed), all chemical shifts of carbon in the headgroup, and part of those in the tail of PHTY, were displaced in the ^13^C-{^1^H} spectra. The C chemical shifts of PT22 (Figure S5) were almost unchanged because the arrangement of lipid molecules is almost stable when the cubic phase is dispersed. Interestingly, during the phase formation, the chemical shifts for PT28 (Figure S6) first exhibited changes, but eventually the chemical shift redirected to the original positions. During the days between days 14 to 63, the additional peaks showed an anisotropic nature (maybe forming a transient L*α* phase). This result suggested that the molecular arrangement of PT28 may migrate during phase formation through a L*α* phase, but eventually form an isotropic cubic phase. This change was not observed in the ^1^H spectra, and the ^13^C-{^1^H} spectra may be more sensitive to the CSA effect.

### 3.4. Intermolecular Interactions by ^1^H-^1^H 2D-NOESY

**Figure 5:**
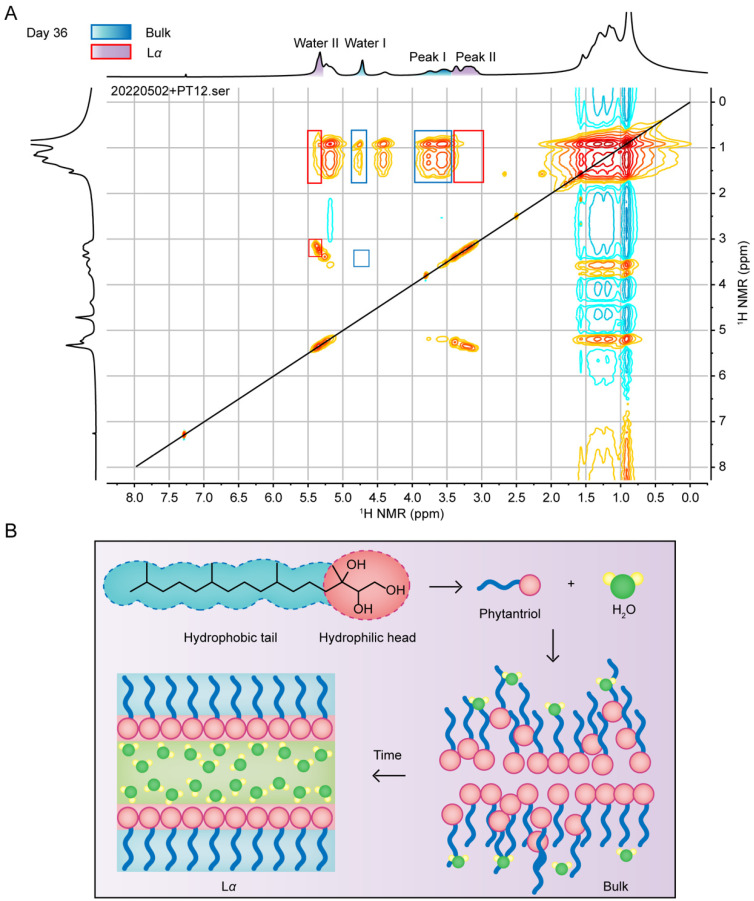
(**A**) A two-dimensional ^1^H-^1^H NOESY spectrum of PT12 on day 36. (**B**) The bulk structure and anisotropic phase model of a phytantriol/water system.

The 2D-NOESY spectrum provides information on the spatial interactions between molecules or within one molecule [27]. The NOESY experiments elucidate interactions between lipid molecules and lipid–water [28,29]. The NOESY spectrum of PT12 during a two-phase coexistence was measured to observe the difference in the interaction of the bulk phase and Lα phase. In Figure 5A, water I, water II, peak I and peak II could be observed in the NOESY spectra. By comparing with NOESY spectra of the first day (Figure S7A) and 90th day (Figure S7B) for the PT12 sample, peak I and peak II represented the characteristics of the bulk phase and Lα phase, respectively. There were no NOE-associated peaks between water I and peak I, while there was a strong correlation (cross peak) between water II and peak II. In addition, the hydrophobic tail of bulk phytantriol has a cross peak with water, while the H of alkane in the tail of the anisotropic phase had no NOE peak with water. Therefore, we speculate that the hydrophilic head of the bulk phytantriol molecule is inward at the beginning, and the water molecules may penetrate the lateral side of the hydrophobic tail. However, when phytantriol self-assembled into an anisotropic layered structure, water molecules gradually moved and distributed in the layer composed of the hydrophilic head (Figure 5B) [9].

**Figure 6:**
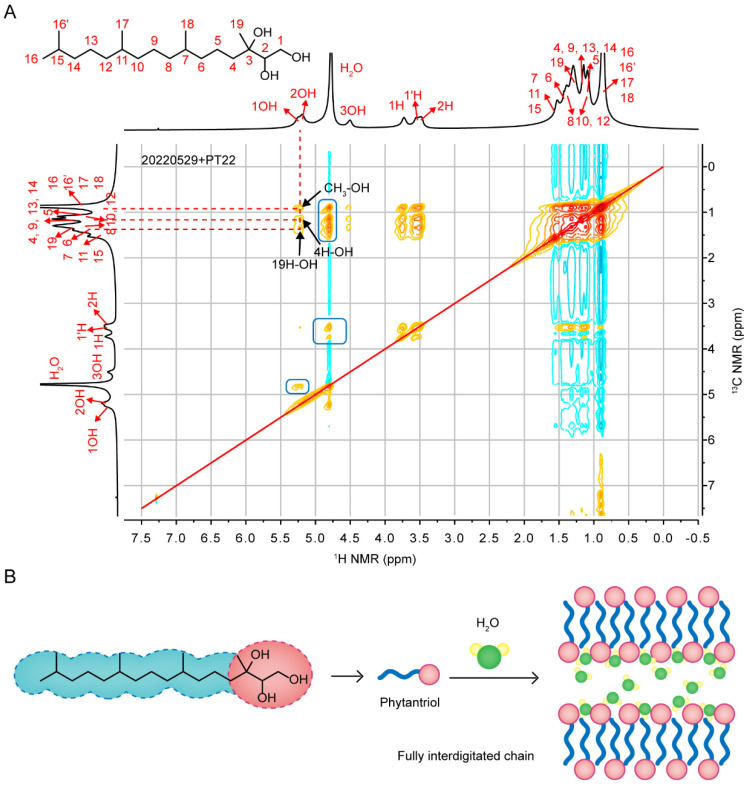
(**A**) The two-dimensional ^1^H-^1^H NOESY spectrum of PT22 on day 63. (**B**) The bulk structure and isotropic phase model of a phytantriol/water system.

We also measured the NOESY spectrum of isotropic PT22. In Figure 6A, hydroxyl H and alkyl H in the hydrophilic head had extensive cross-peaks with H in the hydrophobic tail, and they all had strong NOE effects with the water peak. The reason can be attributed to liquid properties of the lipid in the self-assembled layers. Although water was mainly retained in the hydrophilic channels formed by the hydrophilic heads [30,31,32], the internal lipid arrangement may be compact [10,18,33,34], resulting in spatial interactions both within the heads and tails of the phytantriol and between the heads and tails of the phytantriol and water molecules (Figure 6B) [35].

## 4. Conclusions

In this study, phytantriol-based LLCs with different hydration states were prepared, which belong to different liquid crystal phases (Lα, Q^224^ and Q^230^). We have thoroughly characterized the phytantriol-based LLCs via different NMR techniques. Some new discoveries have been made, which fill the gap in the field of NMR research regarding the phytantriol/water system. First, with the comprehensive analysis of ^1^H, ^13^C-{^1^H}, HSQC and HMBC, the chemical shift of H and C in the phytantriol molecule was clearly assigned in the spectra, especially the complex chemical shifts of the alkane chain. Second, we found the variation of the hydration level during phase formation via long-term NMR measurement. At the ninth week, the samples of different phases reached the theoretical hydration and maintained a stable state, which provided a reference for the formation time of the phytantriol/water binary phase. At the same time, the coexistence of two phases during the transition from the bulk phase to the Lα phase was observed for the first-time using the NMR method. In addition, by analyzing the correlation peaks of two-dimensional NOESY, we infer the phase formation process of the layered anisotropic Lα phase and the isotropic cubic phase, as well as the interaction between lipid molecules and lipid–water molecules during the phase formation [36,37,38,39]. During the formation of the Lα phase, the NOESY spectra reflected the diffusion of water molecules from the lipid tail to the head group, which is the driving force of the self-assembling. During the formation of the Q^224^ phase, the NOESY spectra showed that there were correlation peaks in the head and tail of lipids, as well as between the lipid and water. We speculated that the lipid bilayer of this cubic phase was more closely arranged. This study can be used as a pioneer in the NMR research of phytantriol-based LLCs and provides valuable information for future studies of phytantriol-based nanodrug delivery systems.

## Figures and Tables

**Table 1:** ^1^H and ^13^C-{^1^H} chemical shift attributions of PT22 at 298 k, day 63.

Atoms	^1^H (ppm)	^13^C (ppm)
1	1OH 5.24; 1H 3.72; 1′H 3.55	62.76
2	2OH 5.18; 2H 3.49	77.92
3	3OH 4.50	74.14
4	1.15	28.09
5	1.09	22.78
6	1.43	33.05
7	1.53	28.09
8	1.38	37.92
9	1.15	25.03
10	1.09	37.92
11	1.53	28.09
12	1.09	37.92
13	1.15	27.06
14	1.15	39.59
15	1.53	22.78
16	0.87	22.78
17	0.87	19.80
18	0.87	19.68
19	1.29	25.03

**Table 2:** The resonance integrals of peak I (4.00–3.45 ppm) and peak II (3.45–3.00 ppm).

Time	Integrals	
	Peak I (4.00–3.45 ppm)	Peak II (3.45–3.00 ppm)
1	1	0
14	1	0.03
28	1	0.14
36	1	1.08
42	1	1.82
49	1	1.89
56	1	2.49
63	1	471.66
90	0	1.64

## Data Availability

Not applicable.

## Supplementary Materials

The following supporting information can be downloaded at: https://www.mdpi.com/article/10.3390/pharmaceutics14112312/s1, Table S1: The proportion of NMR samples prepared with different hydration level; Table S2: The NMR experiments and adjusted parameters; Figure S1: (A) ^1^H NMR and (B) ^13^C-{^1^H} NMR spectra of PT22 (22% wt H_2_O) at day 63; Figure S2: ^1^H—^1^H COSY spectrum of PT22 (22% wt H_2_O) at day 63; Figure S3: ^1^H NMR spectra of PT28 changed with time; Figure S4: ^13^C-{^1^H} NMR spectra of PT12 changed with time; Figure S5: ^13^C-{^1^H} NMR spectra of PT22 changed with time; Figure S6: ^13^C-{^1^H} NMR spectra of PT28 changed with time; Figure S7: ^1^H-^1^H 2D-NOESY spectra of PT12 on the (A) day 1 and (B) day 90.
