# Supplementary material for: An NMR Study on Hydration and Molecular Interaction of Phytantriol-Based Liquid Crystals
Source: Pharmaceutics. 2022 Oct 27;14(11):2312. doi: 10.3390/pharmaceutics14112312 (PMC9697928; doi:10.3390/pharmaceutics14112312)
Supplement: Supplementary file 1 [file pharmaceutics-14-02312-s001.zip › pharmaceutics-1957448-supplementary.pdf]

Supplementary file

Table S1. The proportion of NMR samples prepared with different hydration level

Sample name	wt % H_2O	m (phytantriol) mg	V (H_2O) μL
PT12	12	150.5	21
PT22	22	135.6	35
PT28	28	127.1	47
PT80	80	38.5	156

Table S2. The NMR experiments and adjusted parameters.

No.	Spectra name	Acquisition parameters	Processing parameters
10	PROTON	Standard 1D proton	Auto baseline correction method: Polynomial Fit; Auto phase correction; Reference: 7.26 ppm
11	qNMR	Quantitative 1D proton	Same as 10
12	WATERSUP_zgesgppe	1D proton with suppression of H_2O signal	Same as 10
13	C13CPD	Standard 1D 13C	Baseline correction method: Whittaker Smoother; Auto phase correction; Reference: 77.16 ppm
14	HSQCEDETGPSISP	Edited HSQC. 1sw=165, o2p=50	Apodization: Sine square 90; Baseline correction method: Bernstein Polynomial Fit; Auto phase correction
15	PROF_HMBC_WATERSUPP	HMBC with solvent suppression. 1sw=165, o2p=50, ns=4, d1=2, 1td=256	Same as 14
16	NOESYPHPR	NOESY with solvent suppression.	Same as 14
17	COSYGPSW	Standard cosy	Apodization: Sine square 0
18	DOSY_DC	DOSY	

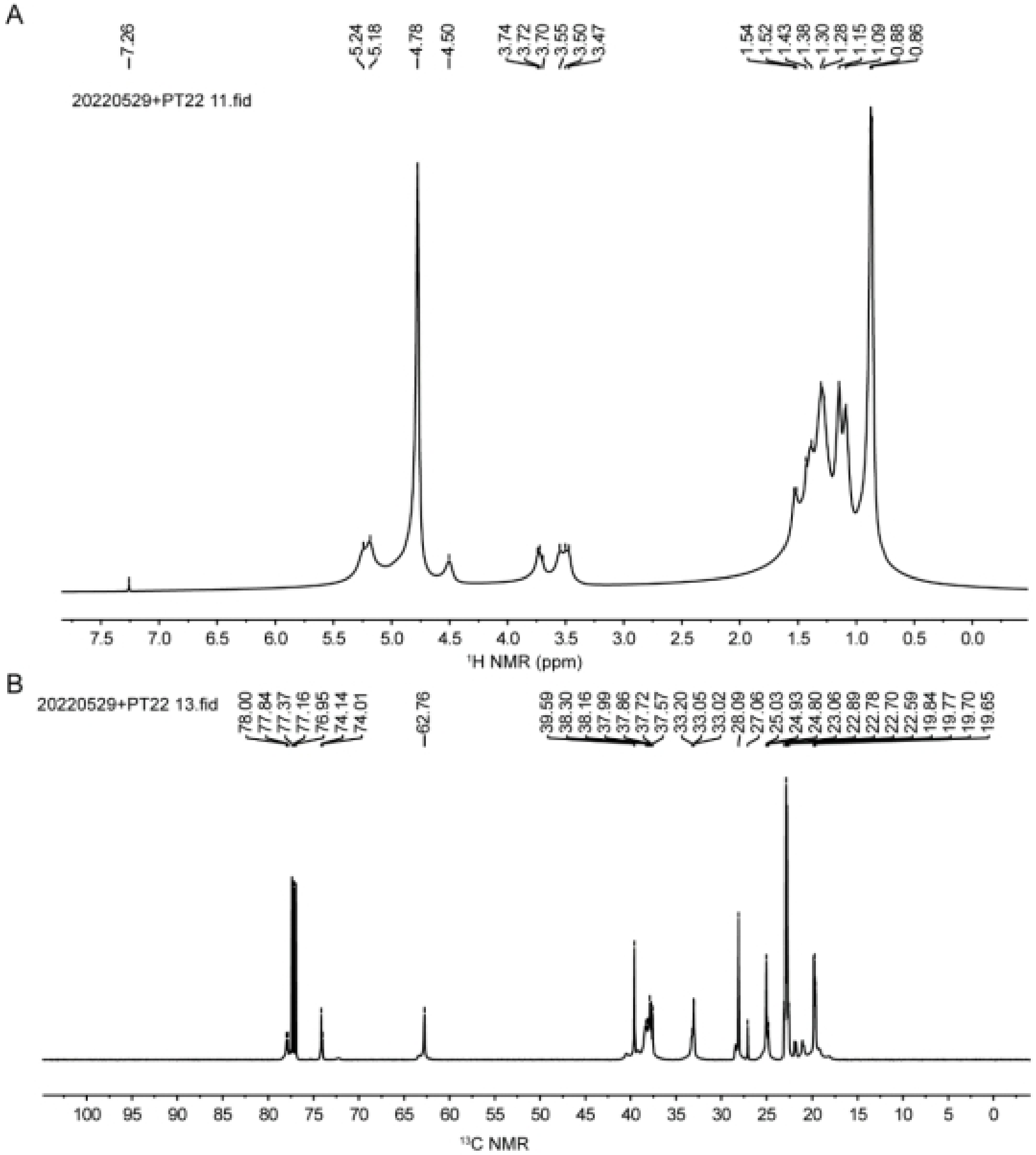

Figure S1. (A) ^1H NMR and (B) ^{13}C NMR spectra of PT22 (22% wt H_2O) at day 63.

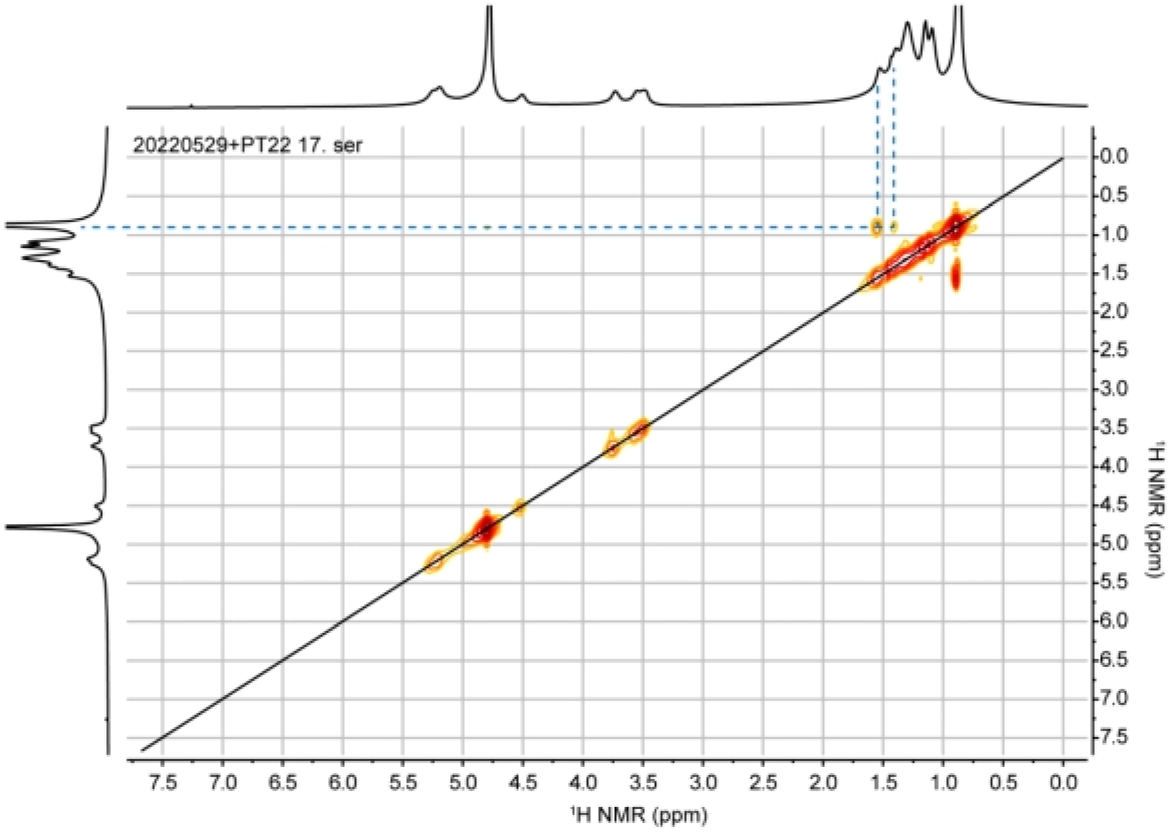

Figure S2. ^{1}H – ^{1}H COSY spectrum of PT22 (22% wt H_2O) at day 63.

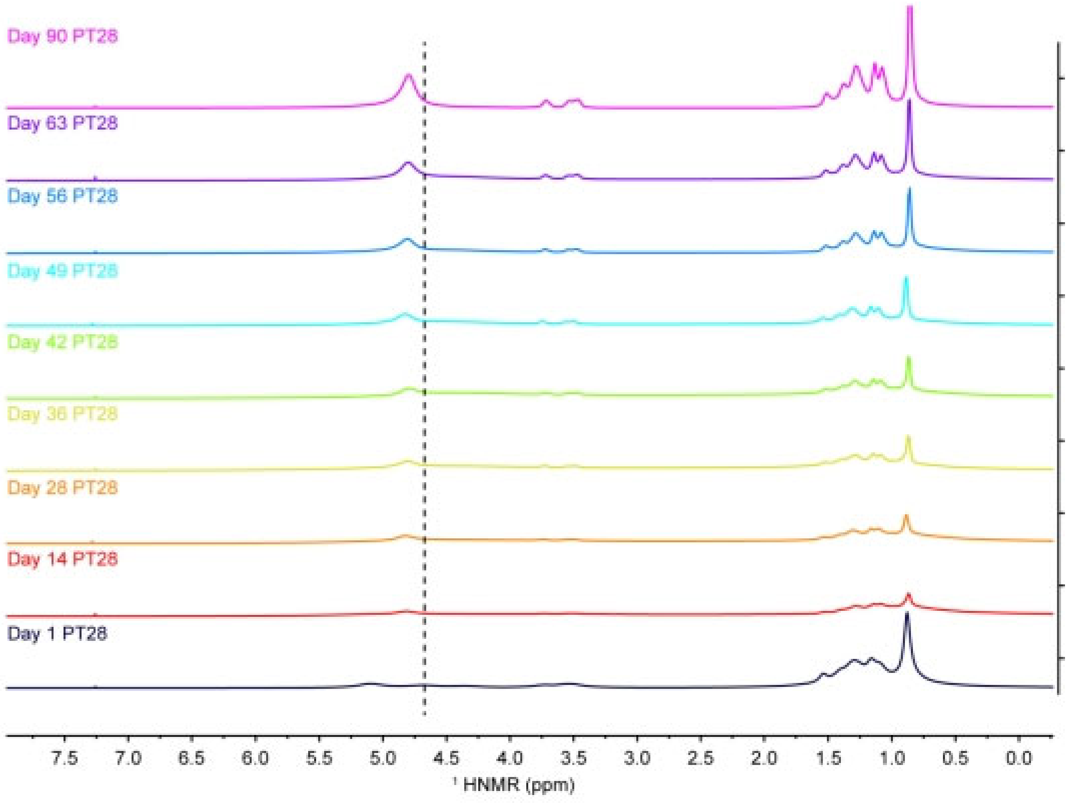

Figure S3. ^{1}H NMR spectra of PT28 changed with time.

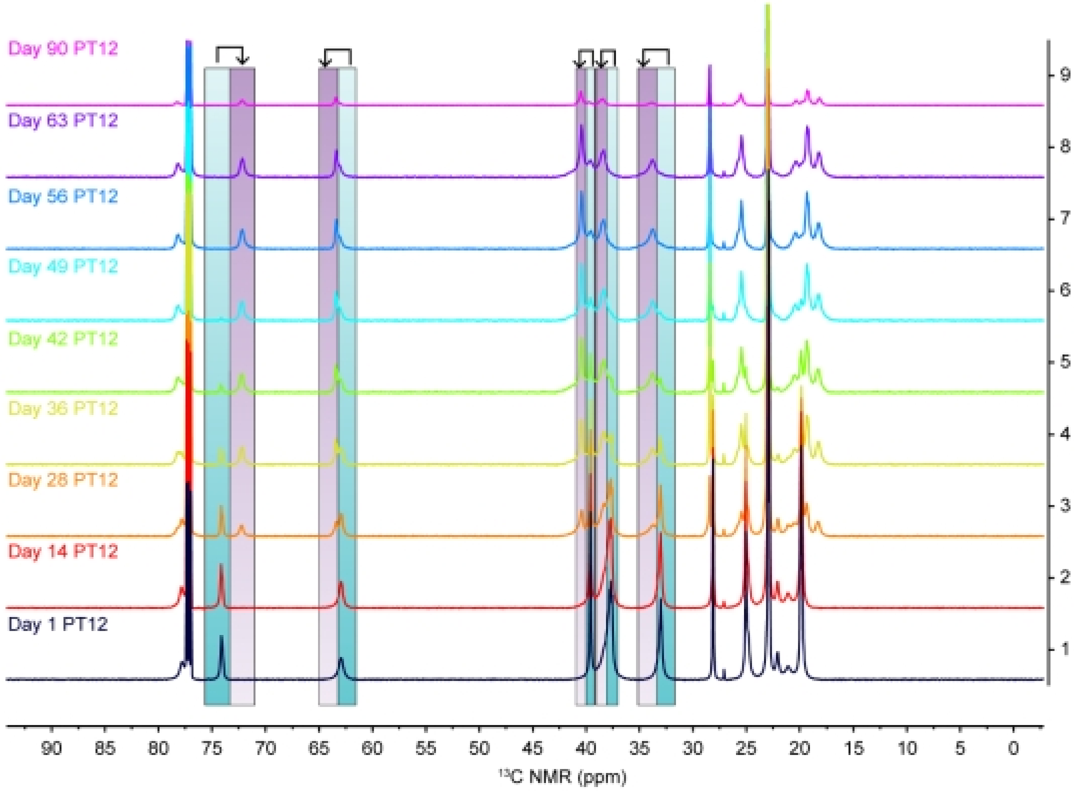

Figure S4. ^{13}C NMR spectra of PT12 changed with time.

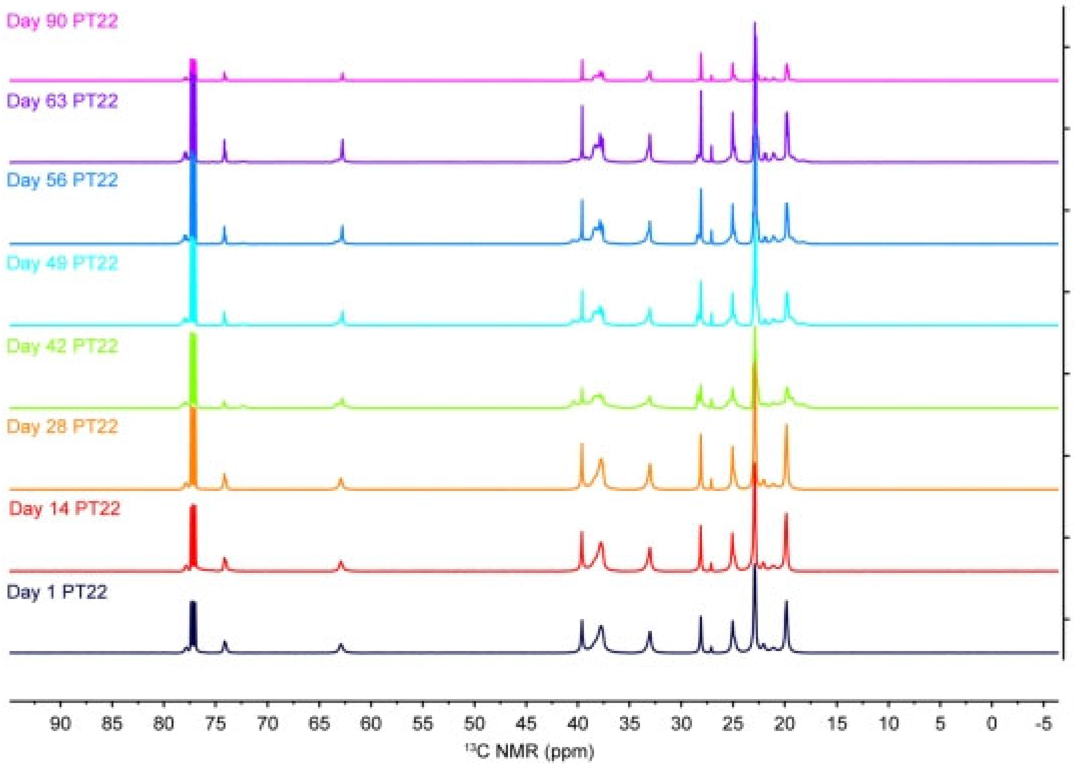

Figure S5. ^{13}C NMR spectra of PT22 changed with time.

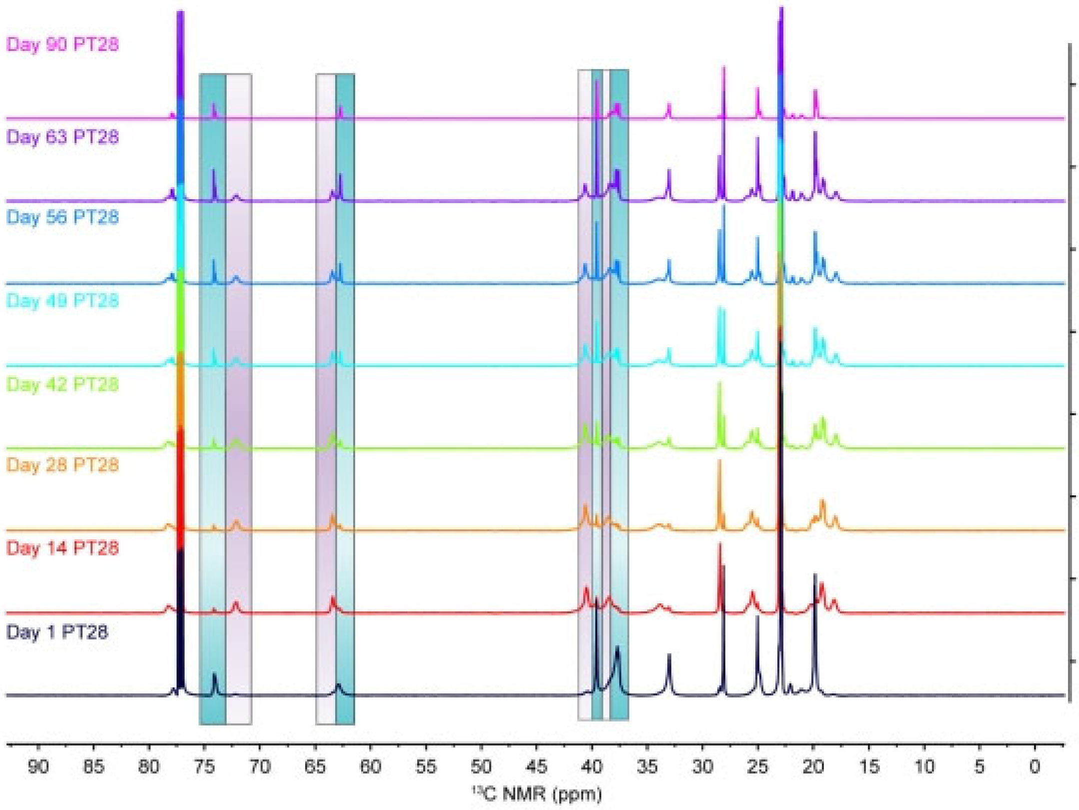

Figure S6. ^{13}C NMR spectra of PT28 changed with time.

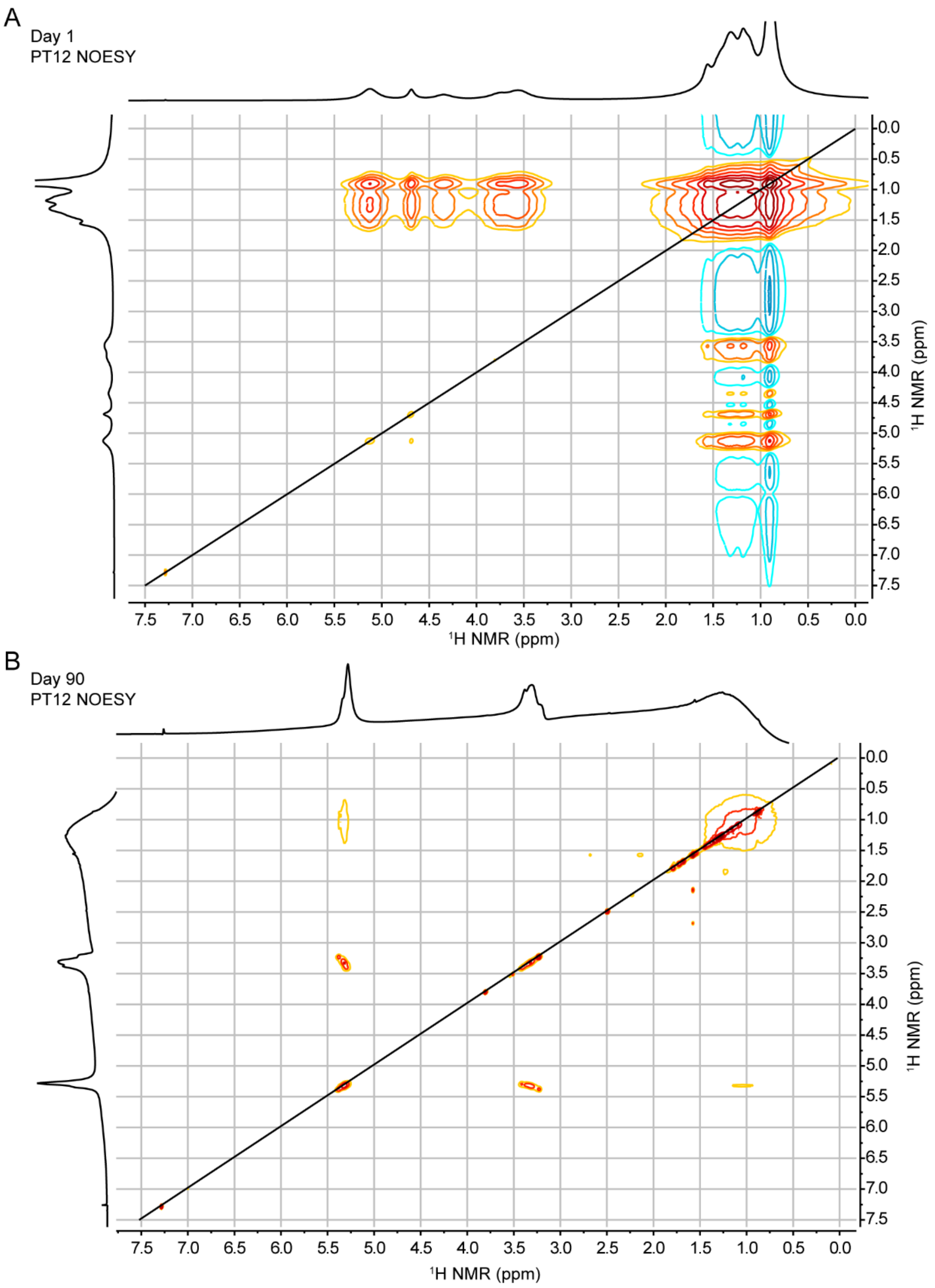

Figure S7. ^{1}H-^{1}H 2D-NOESY spectra of PT12 on the (A) day 1 and (B) day 90.
